# Effect of medical researchers’ creative performance on scientific misconduct: a moral psychology perspective

**DOI:** 10.1186/s12910-022-00876-8

**Published:** 2022-12-18

**Authors:** Na Zhang, Mingxuan Guo, Chunhua Jin, Zhen Xu

**Affiliations:** 1 School of Economics and Management, Beijing Information Science & Technology University, No. 12 Qinghe Xiaoying East Road, Haidian District, Beijing, 100192 China; 2 Medical College, Hebei University of Engineering, Guangming South Street 199, Handan, 056038 Hebei Province China

**Keywords:** Creative performance, Scientific misconduct, Moral licensing, Moral identity

## Abstract

**Background:**

In recent years, some researchers have engaged in scientific misconduct such as fabrication, falsification, and plagiarism to achieve higher research performance. Considering their detrimental effects on individuals’ health status (e.g., patients, etc.) and extensive financial costs levied upon healthcare systems, such wrongdoings have even more salience in medical sciences. However, there has been little discussion on the possible influence of medical researchers’ existing creative performance on scientific misconduct, and the moral psychological mechanisms underlying those effects are still poorly understood.

**Methods:**

We build a moderated mediation model to test how medical researchers’ creative performance affects their scientific misconduct and explore the role of moral licensing and moral identity in this process. Based on situational experiments and projection techniques, 287 medical researchers in China participated in a survey.

**Results:**

Medical researchers’ creative performance positively relates to scientific misconduct, and moral licensing plays a mediating role in the relationship between them. In addition, moral identity has a negative moderating effect on the mediating effect of moral licensing on creative performance and scientific misconduct.

**Conclusion:**

Moral licensing plays a fully mediating role in the relationship between creative performance and scientific misconduct. And moral identity negatively moderates the indirect effect of creative performance on scientific misconduct through moral licensing. The findings provide theoretical and practical implications for the prevention of medical researchers’ scientific misconduct.

**Supplementary Information:**

The online version contains supplementary material available at 10.1186/s12910-022-00876-8.

## Background

As an important means of acquiring and creating knowledge, scientific research has established ethical norms and codes of conduct. However, there have been frequent incidents of scientific misconduct in China and abroad for the last few decades [1]. Considering their detrimental effects on individuals’ health status (e.g., patients, etc.) and extensive financial costs levied upon healthcare systems, such wrongdoings have even more salience in medical sciences [2]. In this context, it is very urgent and important to investigate the factors influencing medical researchers’ scientific misconduct for its prevention. However, the possible influence of medical researchers’ existing creative performance has received little attention in studies of scientific misconduct, and the moral psychological mechanisms underlying those effects are still poorly understood.

Scientific misconduct is unethical behavior such as fabrication, falsification, or plagiarism carried out by researchers during their work [3]. Previous research has indicated that scientific misconduct is vulnerable to individual factors (such as individual characteristics, economic pressure, or excessive pursuit of personal reputation) and environmental factors (such as an imperfect organizational system or unethical academic climate) [4, 5]. Due to the universality and negative effects of scientific misconduct, existing studies have mostly explored the negative affecting factors of scientific misconduct but ignored the positive influencers. Considering the continuity and innovative nature of scientific research, research on the effect of medical researchers’ existing creative performance on their following scientific misconduct is very important, especially for the deep understanding of the causes and processes of scientific misconduct.

Creative performance is defined as engaging in creative behaviors such as suggesting novel and useful products, ideas, or procedures that provide an organization with important raw material for subsequent development and possible implementation [6]. This definition emphasizes that novelty and usefulness are the criteria of creative performance. Existing studies shared the premise that creativity is beneficial for organizations [7, 8], however, the research to unveil the dark side of creativity was called for in the review of creativity literature [9]. Additionally, Gino and Ariely found that creative individuals were more likely to engage in unethical behaviors since they were more capable of justifying their immoral behaviors [8].

Whereas following norms and moral standards requires conformity and convergent thinking, those with high creative performance possess a unique ability to engage in cognitive flexibility [10, 11] and divergent thinking [12]. Consequently, individuals with high creative performance may be more likely to think outside the box in a variety of situations, including those relevant to ethics [13]. Previous studies have found that greater creativity may promote dishonesty in two ways. On the one hand, it can help individuals find creative loopholes to solve difficult tasks they are facing, even if that entails crossing ethical boundaries. On the other hand, creativity may help individuals generate various credible reasons to justify their own actions before engaging in them – even when those actions are unethical [8]. This means researchers who have creative performance may be positively associated with dishonest behavior, such as scientific misconduct [14].

Prior research suggested that people with a high level of creative performance may believe that their creative efforts could contribute to the organization, which may lead them to have a feeling of moral superiority, and thus, they consider themselves moral persons deserving extra preferential treatment [15]. In addition, Zheng et al. found that employees with high creative performance are more likely to engage in workplace deviance [16]. Therefore, creative performance is an important factor that may lead to scientific misconduct. However, the moral psychological mechanism of creative performance influences scientific misconduct needs to be further developed.

The moral licensing theory has often been used to explain why employees change “from good soldiers to bad apples” in the workplace [17, 18], which suggests that people will get a sense of privilege (moral licensing) from past positive behaviors that allow them to subsequently commit unethical behaviors [19]. In other words, moral licensing means that people who have engaged in ethical behavior before will allow themselves to engage in unethical behavior in the future. Moral licensing provides an important theoretical perspective for exploring the psychological mechanism of scientific misconduct induced by creative performance. Although the moral licensing process may be a critical underlying mechanism to explain whether creative performance will allow individuals to break moral standards and further trigger their unethical behavior, current research that links creative performance and moral licensing called for more empirical support [15].

The first aim of this study was to apply moral licensing theory [18] to examine why and how medical researchers’ creative performance may increase scientific misconduct. We propose that medical researchers’ creative performance increases their abilities to justify their potential scientific misconduct. In other words, high creative performance facilitates the self-serving justification process by increasing capacities to develop credible rationalizations for engaging in scientific misconduct [8].

However, this prediction may not be true for all creative performance individuals; those who strive to maintain a positive and honest self-view maybe not susceptible to moral licensing [16]. Individuals vary on moral identity—the centrality of moral traits in one’s overall self-concept [20]—shapes how individuals perceive, form attitudes and react to their ethical stance and actions [21]. Research has shown that individuals with high levels of moral identity tend to enact in accordance with their internal moral standards and in turn behave ethically [22]. Due to its internalized moral self-regulation power, moral identity has been shown to buffer the impacts of certain factors (e.g., depletion) on moral licensing [23]. Prior research has emphasized that moral identity is an important preventive source of undesirable outcomes such as organizational cynicism, workplace silence, and deviance [24].

We conceptualize moral identity as the cognitive schema a person holds about his or her moral character; it is a powerful source of moral motivation because people generally desire to maintain self-consistency[25]. Moral identity reflects the degree of individual recognition of the general moral standards of society and describes the importance of moral values to individuals. Ormiston and Wong proposed that moral identity plays a moderating role in the process of individuals establishing moral licensing [25]. Moral identity, as an important factor that highlights individual differences, may intervene and determine the strength of moral licensing and then affect subsequent behavior [21]. Medical researchers with high moral identity may reduce the occurrence of scientific misconduct by inhibiting the establishment of moral licensing. Therefore, we further proposed that moral identity, a self-view regarding moral traits, is a key lever in determining when creativity performance is associated with moral licensing.

**Fig. 1:**
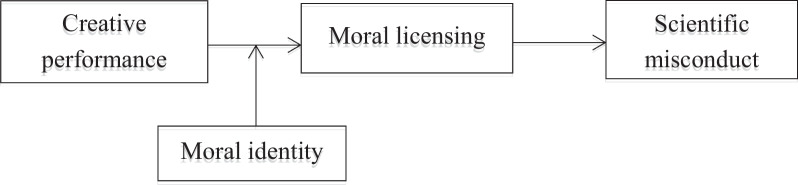
The conceptual model

In summary, this study expands the research on the antecedent variables of scientific misconduct. Starting from the theoretical path of moral licensing, we analyze the impact of medical researchers’ creative performance on scientific misconduct and deeply examine the internal moral psychological mechanism of individuals. We advance the discussion of the influence factors of scientific misconduct beyond the negative side, which is conducive to a more systematic understanding of the antecedents of misconduct in scientific research. At the same time, moral identity, as an individual characteristic that affects individual moral cognition and moral behavior, provides a choice for the formation mechanism of moral cognition of scientific misconduct. Therefore, we examine the moderating role of different degrees of moral identity between medical researchers’ creative performance and moral licensing. Finally, our study reveals the path through which creative performance leads to scientific misconduct and the boundary conditions that affect the path by verifying the moderated mediation model, as shown in Fig. 1. Our findings enrich the theory of scientific misconduct from a moral psychology perspective.

## Methods

### Design and sample

The study was mainly built on a quantitative design and survey research. The random sampling research design was adopted in this study. The participants were medical teachers, postgraduates, and doctoral students who have participated in scientific research from different universities in China. Based on a scenario-based experiment and projection technique, this study set up the situational materials related to the research in advance and presented the corresponding questions from a third-person perspective instead of asking questions directly to the participants. The professional platform “Sojump” was used for collecting the online questionnaire survey. We distributed questionnaires to 350 participants and received 287 valid questionnaires in total (response rate: 88.3%). The sample consisted of 51.8% male and 48.2% female participants. The age distribution was mainly concentrated in the 18–30 years old range, accounting for 63.8% of the total sample. The education level of the subjects was mainly concentrated among postgraduate students and doctoral students, accounting for 94.7% of the total sample.

### Scenario-based experiment and projection technique

We adopted the scenario-based experiment method and psychological projection techniques. The core of scenario simulation is to present the participants with stimulating materials with unclear meaning or not directly related to them and infer their true attitude and judgment by observing their response. Previous psychological studies have shown that projection technology can bypass the resistance and avoidance of participants at the level of consciousness, and it is suitable for understanding the participants’ true attitudes towards sensitive topics [3]. Unlike questionnaires that directly ask participants for their opinions and attitudes, the core of scenario simulation is to provide respondents with a carefully crafted description of a scenario that reflects the real situation, and then indirectly ask them for their attitudes based on the characteristics of the scenario. The scenario simulation method can improve the sense of presence and reduce the social desirability of the participants, so it is widely used in the research of individual attitudes, behavioral intentions, and decision-making [26]. This study also adopted this method.

In order to better compare the differences, the questionnaire adopts the method of within-group design. Each participant will answer questions at two levels of creativity performance, one of which the medical researcher is set as a person with high creativity performance, and the other is set as a person with low creativity performance. At the beginning of the questionnaire, there is a description of the performance level of the medical researcher, as shown in the Additional file 1. The participants need to answer the questionnaires under the two creativity performance levels respectively, and the two questionnaires have the same questions. The order in which the creativity performance levels appeared was random, and the participants were randomly drawn to the questionnaires in different orders.

### Measures

We followed the order in which the measurements are presented in the questionnaire.

According to Bai et al.’s [3] research, scientific misconduct was adapted on the basis of their scale according to the characteristics of the subjects in this paper. As shown in Table 1, the scenarios included four kinds of scientific misconduct, such as misuse of funds, false signature, repeated declaration, and false project marking. We asked three questions for each situation to test the participants’ scientific misconduct from the perspectives of acceptance, consistency, and uniformity. Through measuring participants’ judgments of scientific misconduct by medical researchers with different creative performances, it reflects their own attitudes towards scientific misconduct. Cronbach’s *α* for the whole scale of scientific misconduct was 0.788.

**Table 1:** Scenarios of scientific misconduct

Scientific misconduct	Scenarios
Misuse of funds	During the reimbursement process of project funds, considering the physical and mental efforts he had taken participating in the project, the medical researcher reported some amounts that exceeded the actual expenses.
False signature	The medical researcher is applying for a scientific research project that is jointly participated by several scholars in the institution. When submitting the paper application form, he arranged others to sign the application form for some collaborators without notifying them.
Repeated declaration	As the medical researcher was successfully approved for a major national project last year, this year, he arranged a core member of his project team to submit a proposal to another research program based on the main content of that approved project, in order to obtain more research funding.
False project marking	A scientific research project undertaken by the medical researcher is about to end, and it is found that there is a gap between the research results and the expected goals in the project declaration. He asked the project team members to mark this funding number in all papers and other results, even if some papers are not related to this project. In addition, he also planned to add the results of other projects that are of little concern when writing the final report to ensure a smooth conclusion.

To measure creative performance, we adopted the three-item scale developed by Zhou and George [27]. Participants need to read the profile description of the creative performance level before answering questions and making judgments. The measure was manipulated to ensure that the participants’ answers were valid. Cronbach’s *α* for the whole scale of creative performance was 0.854.

According to the suggestions of Yam et al. [18], we used psychological entitlement as an alternative to measuring the role of moral licensing [28]. Six questions suitable for this test were selected and adapted according to the situation. In order to measure the role of moral licensing, the questionnaire asked the participants to think about their choices on the previous items: “You made this judgment because: …” Cronbach’s *α* for the whole scale of moral licensing was 0.872.

Moral identity was measured using the 10-item scale developed by Aquino and Reed [20]. They divided the scale into two dimensions: internalization and symbolization. Considering that if moral identity is measured at the beginning or in the middle, the positiveness of the items may interfere with other scales, so it is set to at the end of the questionnaire. Cronbach’s *α* for the whole scale of moral identity was 0.702.

### Data analysis

SPSS 25.0 and the SPSS macro program PROCESS by Hayes [29] were utilized for data analysis. PROCESS can verify a variety of moderated mediation models based on the bootstrap method of deviation correction percentile. By sampling 5000 bootstrap samples, the robust standard error and bootstrap confidence interval of parameter estimation were obtained. If the confidence interval did not contain 0, the result was statistically significant.

## Results

### Common method variance test

The present study used self-reports to assess all variables that may have common method variance problems to influence the results. The questionnaire of this study was controlled from the perspective of the third person. Meanwhile, the common method variance was tested by a Harman single-factor test. The results showed that there are seven factors with characteristic roots greater than 1, and the cumulative variation explained by the first factor was only 29.86% (less than 40%), indicating that there was no serious common method variance in this study [30].

### Descriptive statistics

Table 2 details the means, standard deviations, and intervariable correlations of all variables. The results showed that creative performance was significantly positively correlated with scientific misconduct, creative performance was significantly positively correlated with moral licensing, moral licensing was significantly positively correlated with scientific misconduct, and moral identity was significantly negatively correlated with moral licensing and scientific misconduct.

**Table 2:** Pearson’s correlation coefficient values

Variable	M	SD	1	2	3	4	5	6	7
1Gender	1.48	0.50	1						
2Age	2.16	0.91	−0.232**	1					
3Education level	3.02	0.40	−0.078	0.264**	1				
4Scientific misconduct	4.36	0.61	−0.064	−0.081	0.01	1			
5Creative performance	5.07	1.17	−0.018	0.005	0.075	0.237**	1		
6Moral licensing	4.31	1.21	−0.124*	−0.009	0.041	0.278**	0.547**	1	
7Moral identity	4.44	0.54	0.031	0.083	0.014	−0.149*	−0.265**	−0.608**	1

*p < 0.05; **p < 0.01; ***p < 0.001

### Hypothesis testing

An independent *t* test was used to analyze the difference between the high creative performance group (M = 4.36, SD = 0.61) and the low creative performance group (M = 3.95, SD = 0.73) in the scientific misconduct score. The scores of the two groups were significantly different, *t* (488.8) = 6.8, *p* < 0.001, *d* = 0.61. Compared with those with low creative performance, the scientific misconduct scores of medical researchers with high creative performance were higher, which preliminarily verifies the hypothesis that creative performance is positively related to scientific misconduct.

Model 4 in PROCESS was used to test the mediating effect of moral licensing between the creative performance of medical researchers and scientific misconduct. As seen in Table 3 showed that creative performance was positively related to moral licensing (*coefficient* = 0.56, *p* < 0.05), and moral licensing was positively related to scientific misconduct with the addition of a mediating variable (*coefficient* = 0.13, *p* < 0.01).

**Table 3:** Mediating effect analysis

		Effect	SE	*p*	Confidence interval		R^2^	F
					LLCI	ULCI		
Moral licensing	Constant	1.45	0.27	0	0.92	1.98	0.29	11
	Creative performance	0.56	0.05	0	0.46	0.67		
Scientific misconduct	Constant	3.24	0.21	0	2.82	3.66	0.09	13.38
	Moral licensing	0.13	0.05	0.2*	0.05	0.23		
	Creative performance	0.08	0.05	0.07	−0.01	0.17		

*p < 0.05; **p < 0.01; ***p < 0.001

As seen in Table 4, the bootstrap 95% confidence interval of the total effect of creative performance on scientific misconduct and the mediating effect of moral licensing did not contain 0. After adding the mediating variable of moral licensing, the bootstrap 95% confidence interval of the direct effect of creative performance on scientific misconduct contained 0. Therefore, moral licensing fully mediated the relationship between creative performance and scientific misconduct, and the mediating effect (0.074) accounted for 46% of the total effect (0.159).

**Table 4:** Analysis of direct and indirect effects

	Effects	Boot SE	Boot LLCI	Boot ULCI
Total effect	0.159	0.039	0.081	0.237
Direct effect	0.084	0.046	−0.007	0.176
Mediating effect	0.074	0.027	0.020	0.127

Next, Model 7 in PROCESS was used to test the moderating effect of moral identity. The results in Table 5 showed that the interaction between creative performance and moral identity had a significant impact on moral licensing (*coefficient* = − 0.36, *p* < 0.01,95%CI = [−0.53, −0.19]). In other words, moral identity negatively moderated the relationship between creative performance and moral licensing. When the level of moral identity was low (M − SD), the mediating effect value of moral licensing was 0.08, 95%CI = [0.02, 0.15]; when the moral identity level was high (M + SD), the mediating effect value of moral license was reduced to 0.03, 95%CI = [0.00, 0.06]. This showed that moral identity negatively moderated the mediating effect of moral licensing on the relationship between creative performance and scientific misconduct.

**Table 5:** Moderating effect analysis

	Effect	SE	*p*	LLCI	ULCI
Creative performance	2.07	0.4	0	1.28	2.85
Moral identity	0.77	0.46	0.09	−0.14	1.69
Creative performance × moral identity	−0.36	0.09	0	−0.53	−0.19

*p < 0.05; **p < 0.01; ***p < 0.001.

**Fig. 2:**
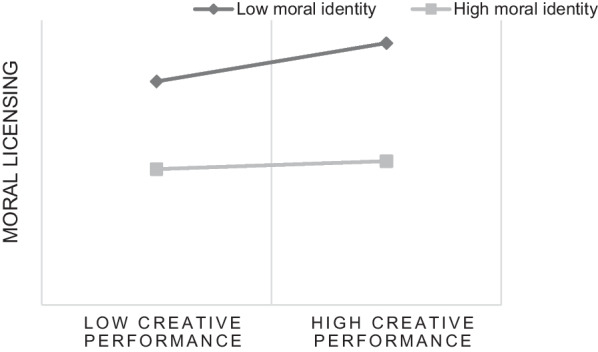
Creative performance × Moral identity interaction for moral licensing

To further explain the essence of the interaction between creative performance and moral identity more clearly, we divided moral identity into high and low groups according to the average addition and subtraction of a standard deviation (Fig. 2). A simple slope test showed that for medical researchers with low moral identity, the creative performance had a significant positive impact on moral licensing (*B*_simple_ = 0.65, *t* = 9.45, *p* < 0.01), while for medical researchers with high moral identity, the positive impact of creative performance on moral licensing was weakened (*B*_simple_ = 0.26, *t* = 4.61, *p* < 0.001; *B*_simple_ = 0.65 reduced to *B*_simple_ = 0.26).

## Discussion

### Major research findings

This study investigated the influence of creative performance, a positive behavior, on the scientific misconduct of medical researchers, which is a useful attempt to explore the influence of positive factors on scientific misconduct. By clarifying the psychological mechanism of individual moral licensing, we discuss the effect process of medical researchers’ creative performance on scientific misconduct. Our findings expand the research perspective on the influencing factors of scientific misconduct and enrich the research on the dark side effects of creativity. At the same time, this study provides a theoretical reference and useful information for the management of medical research institutions to prevent the occurrence of scientific misconduct and its negative impact.

The theoretical significance of this paper is as follows. First, we found that creative performance could lead medical researchers to commit scientific misconduct, effectively expanding the research on the dark side of creativity. The existing research on creative performance has focused on exploring the influencing factors of creativity from two perspectives: individual characteristics [31] and situational factors [32]. Although the focus of these studies was different, there was a common potential premise: individual creative performance improves problem-solving ability and makes it possible to discover new solutions and opportunities. In other words, creative performance is always beneficial. However, our research examined the negative influence of creative performance. We found that medical researchers with high creative performance could also bring negative influences such as scientific misconduct. The results enrich the research on the dark side of creative performance and provide new ideas for follow-up research.

Second, based on the path of moral licensing, this study examined the psychological mechanism of the effect of creative performance on scientific misconduct. The possible explanation is that medical researchers with high creative performance could establish moral licensing considering their previous contributions to the organization, resulting in subsequent scientific misconduct. Most of the existing pieces of literature on research ethics are discussed from the perspective of phenomenon description or philosophical speculation, and there is a lack of deep discussion on the psychological mechanism before the emergence of individual research misconduct. However, most of the existing research results on the influencing factors of research misconduct focus on the influence of negative factors (such as moral decay and low quality, imperfect organizational system, etc.) [33, 34], ignoring that positive factors may also lead to the occurrence of misconduct in scientific research. Moral licensing could explain why employees change “from good soldiers to bad apples” in the workplace. Therefore, based on the moral licensing theory, this study examined the effect of medical researchers’ creative performance on scientific misconduct and described the psychological mechanism in detail, which is a further expansion of the research on the influencing factors of scientific misconduct.

Third, our findings enrich the boundary conditions of the inhibition mechanism of scientific misconduct. We highlighted that creativity performance was not always associated with scientific misconduct; instead, individual differences such as moral identity might influence the consequences of creativity performance. Moral identity not only moderates the relationship between creative performance and moral licensing but also moderates the mediating effect of creative performance on scientific misconduct. The results show that, compared with medical researchers with low moral identity, individuals with high moral identity are more likely to restrain the establishment of moral licensing. Therefore, when the medical researcher’s moral identity level is high, creative performance may weaken their moral licensing level and then restrain the occurrence of scientific misconduct. Our research not only further clarifies the boundary conditions of the inhibition mechanism of scientific misconduct but also provides a theoretical basis for medical research institutions to avoid the continued occurrence of scientific research misbehavior.

### Implications for management

As medical research institutions strive to decrease medical researchers’ scientific misconduct, our findings provided several important implications for management practices. First, managers should understand the two sides of creative performance. In daily management, managers should pay more attention to the medical researchers’ creative performance and guide them accordingly. Especially when medical researchers with low moral identity have high creative performance, managers should timely intervene, strengthen communication with them, and reduce the possibility of their moral licensing and in turn, reduce the possibility of their scientific misconduct.

Second, organizations should increase their focus on medical researchers’ moral identity. On the one hand, organizations should take moral identity as an important selection indicator when recruiting, and conduct assessments through questionnaires and in-depth interviews, so as to select medical researchers with high moral identity; On the other hand, organizations should improve medical researchers’ moral identity by formulating ethical norms and cultivating ethical climate. Especially, organizations can consider launching training programs to foster the development of moral identity.

### Limitations

It is important to consider the limitations of this study when interpreting the results.

First, this study examined the path of moral licensing; however, to date, there is no mature scale that can be used to measure moral licensing. Some scholars have supported the use of 10 questions about moral credits developed by Lin [35]. This is a good starting point for the development of a moral licensing scale. Although some relevant experts have put forward guiding opinions, scales have only been developed theoretically and not applied in a practical investigation, so more research is needed to combine the relevant theories into a mature scale. In addition, for the research in China, the development of the scale needs to be combined with the national conditions, such as taking into account the moral orientation in the Chinese cultural environment.

Second, the research took medical researchers as the object of study, and more perfect classification criteria can be used in future research, such as focusing on researchers who have participated in major medical research projects in scientific research institutions, or focusing on the measurement of medical teachers, postgraduates, doctoral students and other groups, according to different levels of researchers, there may be more research results for reference. At the same time, based on the new findings obtained in this study, future research can be classified and studied in terms of the severity of misconduct in medical research to get more abundant results.

## Conclusion

From a moral psychology perspective, this study took moral licensing as a mediating variable and moral identity as a moderating variable to construct a moderated mediation model to explore the impact of medical researchers’ creative performance on scientific misconduct. The results show that moral licensing plays a complete mediating role in the relationship between creative performance and scientific misconduct. The higher the level of creative performance, the more likely medical researchers are to engage in moral licensing, and they then have a higher likelihood of engaging in scientific misconduct. In addition, moral identity negatively moderates the indirect effect of creative performance on scientific misconduct through moral licensing. These findings provide inspiration and practical significance for the prevention of scientific misconduct in medical research institutions.

## Supplementary Information

**Additional file 1.** This file contains the questionnaire used in this study.

## Data Availability

The datasets generated and analyzed during the current study are not publicly available but are available from the corresponding author on reasonable request.
