# Supplementary material for: Effect of medical researchers’ creative performance on scientific misconduct: a moral psychology perspective
Source: BMC Med Ethics. 2022 Dec 18;23:137. doi: 10.1186/s12910-022-00876-8 (PMC9759886; doi:10.1186/s12910-022-00876-8)
Supplement: Supplementary file 1 — Additional file 1. This file contains the questionnaire used in this study. [file 12910_2022_876_MOESM1_ESM.docx]

**PRAT 1**

***A medical researcher has participated in several major research projects and won considerable honors last year. He published many academic articles and achieved excellent scientific results. He had an extraordinary performance in the research institution, so he has gained a lot of social praise and credibility.***

1. The following are four kinds of behaviors that the **high-performing** medical researcher may exhibit during the project submission and implementation process in this year. Each scenario is accompanied by three corresponding questions. Please make a judgment based on your understanding of the medical researcher’s behavior in the material.

**Scenario 1**: During the reimbursement process of project funds, considering the physical and mental efforts he had taken participating in the project, the medical researcher reported some amounts that exceeded the actual expenses.

1. Do you accept of the behavior by the medical researcher described in the contextual material?

Completely unacceptable 1—2—3—4—5—6—7 Completely accepted

1. Do you think the researcher has done this behavior in previous projects?

Never 1—2—3—4—5—6—7 Consistently

1. Do you think the medical researcher will do this kind of behavior in other projects in the future?

Definitely not 1—2—3—4—5—6—7 Definitely will

**Scenario 2**: The medical researcher is applying for a scientific research project that is jointly participated by several scholars in the institution. When submitting the paper application form, he arranged others to sign the application forms for some collaborators without notifying them.

1. Do you accept of the behavior by the medical researcher described in the contextual material?

Completely unacceptable 1—2—3—4—5—6—7 Completely accepted

1. Do you think the researcher has done this behavior in previous projects?

Never 1—2—3—4—5—6—7 Consistently

1. Do you think the medical researcher will do this kind of behavior in other projects in the future?

Definitely not 1—2—3—4—5—6—7 Definitely will

**Scenario 3**: As the medical researcher was successfully approved for a major national project last year, this year, he arranged a core member of his project team to submit a proposal to another research program based on the main content of that approved project, in order to obtain more research funding.

1. Do you accept of the behavior by the medical researcher described in the contextual material?

Completely unacceptable 1—2—3—4—5—6—7 Completely accepted

1. Do you think the researcher has done this behavior in previous projects?

Never 1—2—3—4—5—6—7 Consistently

1. Do you think the medical researcher will do this kind of behavior in other projects in the future?

Definitely not 1—2—3—4—5—6—7 Definitely will

**Scenario 4**: A scientific research project undertaken by the medical researcher is about to end, and it is found that there is a gap between the research results and the expected goals in the project declaration. He asked the project team members to mark this funding number in all papers and other results, even if some papers are not related to this project. In addition, he also planned to add the results of other projects that are of little concern when writing the final report to ensure a smooth conclusion.

1. Do you accept of the behavior by the medical researcher described in the contextual material?

Completely unacceptable 1—2—3—4—5—6—7 Completely accepted

1. Do you think the researcher has done this behavior in previous projects?

Never 1—2—3—4—5—6—7 Consistently

1. Do you think the medical researcher will do this kind of behavior in other projects in the future?

Definitely not 1—2—3—4—5—6—7 Definitely will

1. Please recall the description at the beginning of the survey about the **high-performing** medical researcher in the scenario and make a judgment: (From 1 = completely disagree to 7 = completely agree)

(1) The medical researcher often has new and innovative ideas.

(2) The medical researcher often comes up with new and practical ideas to improve scientific research performance.

(3) This medical researcher is a good source of creative ideas in scientific institutions.

1. You made these judgments about the above four behaviors of the medical researcher because (From 1 = completely disagree to 7 = completely agree)
2. I honestly feel he’s just more deserving than others.
3. He is entitled to more right.
4. The progress of scientific research projects should be developed in accordance with his way.
5. Major scientific research projects should be led by him.
6. He demands the best because he’s worth it.
7. People like him deserve an extra break now and then.

**PRAT 2**

*A medical researcher at a research institution had poor research performance last year, and even failure to meet the performance appraisal goals. Compared with colleagues who have participated in many major projects and achieved excellent scientific research results, the medical researcher lacks scientific research ability and confidence.*

1. The following are four kinds of behaviors that the **low-performing** medical researcher may exhibit during the project submission and implementation process in this year. Each scenario is accompanied by three corresponding questions. Please make a judgment based on your understanding of the medical researcher’s behavior in the material.

**Scenario 1**: During the reimbursement process of project funds, considering the physical and mental efforts he had took participating in the project, the medical researcher reported some amounts that exceeded the actual expenses.

1. Do you accept of the behavior by the medical researcher described in the contextual material?

Completely unacceptable 1—2—3—4—5—6—7 Completely accepted

1. Do you think the researcher has done this behavior in previous projects?

Never 1—2—3—4—5—6—7 Consistently

1. Do you think the medical researcher will do this kind of behavior in other projects in the future?

Definitely not 1—2—3—4—5—6—7 Definitely will

**Scenario 2**: The medical researcher is applying for a scientific research project that is jointly participated by several scholars in the institution. When submitting the paper application form, he arranged others to sign the application forms for some collaborators without notifying them.

1. Do you accept of the behavior by the medical researcher described in the contextual material?

Completely unacceptable 1—2—3—4—5—6—7 Completely accepted

1. Do you think the researcher has done this behavior in previous projects?

Never 1—2—3—4—5—6—7 Consistently

1. Do you think the medical researcher will do this kind of behavior in other projects in the future?

Definitely not 1—2—3—4—5—6—7 Definitely will

**Scenario 3**: As the medical researcher was successfully approved for a major national project this year, he arranged a core member of his project team to submit a proposal to another research program based on the main content of that approved project, in order to obtain more research funding.

1. Do you accept of the behavior by the medical researcher described in the contextual material?

Completely unacceptable 1—2—3—4—5—6—7 Completely accepted

1. Do you think the researcher has done this behavior in previous projects?

Never 1—2—3—4—5—6—7 Consistently

1. Do you think the medical researcher will do this kind of behavior in other projects in the future?

Definitely not 1—2—3—4—5—6—7 Definitely will

**Scenario 4**: A scientific research project undertaken by the medical researcher is about to end, and it is found that there is a gap between the research results and the expected goals in the project declaration. He asked the project team members to mark this funding number in all papers and other results, even if some papers are not related to this project. In addition, he also planned to add the results of other projects that are of little concern when writing the final report to ensure a smooth conclusion.

1. Do you accept of the behavior by the medical researcher described in the contextual material?

Completely unacceptable 1—2—3—4—5—6—7 Completely accepted

1. Do you think the researcher has done this behavior in previous projects?

Never 1—2—3—4—5—6—7 Consistently

1. Do you think the medical researcher will do this kind of behavior in other projects in the future?

Definitely not 1—2—3—4—5—6—7 Definitely will

1. Please recall the description at the beginning of the survey about the **low-performing** medical researcher in the scenario and make a judgment: (From 1 = completely disagree to 7 = completely agree)

(1) The medical researcher often has new and innovative ideas.

(2) The medical researcher often comes up with new and practical ideas to improve scientific research performance.

(3) This medical researcher is a good source of creative ideas in scientific institutions.

1. You made these judgments about the above four behaviors of the medical researcher because (From 1 = completely disagree to 7 = completely agree)
2. I honestly feel he’s just more deserving than others.
3. He is entitled to more right.
4. The progress of scientific research projects should be developed in accordance with his way.
5. Major scientific research projects should be led by him.
6. He demands the best because he’s worth it.
7. People like him deserve an extra break now and then.

**PRAT 3**

Here are some words that describe a person’s traits: caring, compassionate, fair, friendly, generous, helpful, hardworking, honest, and kind. Please judge the degree of conformity of each of the following statements. (From 1 = strong disagreement to 7 = strong agreement)

1. It would make me feel good to be a person who has these characteristics.
2. Being someone who has these characteristics is an important part of who I am.
3. I often wear clothes that identify me as having these characteristics.
4. I would be ashamed to be a person who has these characteristics.
5. The types of things I do in my spare time (e.g., hobbies) clearly identify me as having these characteristics.
6. The kinds of books and magazines that I read identify me as having these characteristics.
7. Having these characteristics is not really important to me.
8. The fact that I have these characteristics is communicated to others by my membership in certain organizations.
9. I am actively involved in activities that communicate to others that I have these characteristics.
10. I strongly desire to have these characteristics.
